# Determinants of Exclusive Breastfeeding Practices Among Mothers of Infants Less Than Six Months Attending an Immunization Clinic in Southwestern Nigeria

**DOI:** 10.7759/cureus.15975

**Published:** 2021-06-27

**Authors:** Yetunde T Olasinde, Olayinka R Ibrahim, Ajibola Idowu, Abimbola O Odeyemi, Adeola Olasinde, Efeturi Agelebe, Olumuyiwa A Ogunlaja, Daniel A Gbadero

**Affiliations:** 1 Paediatrics and Child Health, Bowen University, Iwo, NGA; 2 Paediatrics, Federal Medical Center, Katsina, NGA; 3 Epidemiology and Public Health, Bowen University, Iwo, NGA; 4 Obstetrics and Gynecology, Civil Service Hospital, Ilorin, NGA; 5 Obstetrics and Gynecology, Bowen University, Iwo, NGA

**Keywords:** exclusive breastfeeding, ogbomoso nigeria, immunisation clinic, infant and young child feeding practices, infants less than six months

## Abstract

Background

Despite being a cost-effective means of improving the childhood health indices, exclusive breastfeeding (EBF) remains low in the low middle-income countries. Hence, we evaluated the determinants of EBF among mothers of infants less than six months in Southwestern Nigeria.

Methods

This was a cross-sectional descriptive study that involved 271 mothers of infants aged less than six months attending the immunization clinic of the Bowen University Teaching Hospital, Ogbomoso, Nigeria. Pretested semi-structured questionnaires were used to get relevant information from the mothers who were recruited using convenience sampling method. Descriptive statistics was carried out while chi square test and binary logistic regression were used for inferential statistics.

Results

The mean age (±SD) of the respondents was 30.4 ± 5.0 years. The EBF rate in this study was 46.1% (125/271); 40.6% of mothers breastfed their infants within an hour of birth, with most (91.1%) breastfeeding their babies on demand. Factors associated with EBF included mothers’ age > 30 years (OR 2.080, 95% CI 1.274-3.395). After controlling for potential confounders, family size > 4, (adjusted OR 2.053, 95% CI 1.120-3.762) and having vaginal delivery (adjusted OR 2.769, 95% CI 1.585-4.829) were the significant determinants of EBF practices among the study participants.

Conclusion

EBF practice was average in the studied population. Family size >4 and vaginal delivery were the determinants of EBF. There is a need to sustain the promotion of appropriate breastfeeding practices.

## Introduction

Breastfeeding remains a key strategy for improving public health with benefits to infants, mothers, and the community at large. The World Health Organization (WHO) and United Nations Children's Fund (UNICEF) recommend early initiation of breastfeeding, exclusive breastfeeding during the first six months of life, and continued breastfeeding until 24 months of age [1, 2]. Exclusive breastfeeding (EBF), defined as the feeding of infants with only human milk (either directly or expressed) for the first six months of life, is the most cost-effective means of reducing childhood morbidity and mortality in the low middle-income countries (LMICs) [3, 4]. Infants who are partially breastfed and those who are not breastfed have 2.3- and 2.5-fold increased risk of mortality, respectively [5]. Besides, delays in the initiation of breastfeeding after birth could be harmful for the newborns. Studies show that newborns with delayed initiation of breastfeeding between 2 and 23 hours after birth have as high as 33% increased risk of dying [6, 7].

Despite the WHO and UNICEF recommendations, breastfeeding practice rates remain low, with only two out of five newborns being breastfed within an hour of birth [6]. Only 40% of infants aged six months or less are exclusively breastfed [1, 4]. In the LMICs, including Nigeria, exclusive breastfeeding rates are low [8-11]. The 2018 Nigeria Demographic and Health Survey (NDHS) showed that the exclusive breastfeeding rate in Nigerian children aged less than six months was 28.7% [12].

Studies outside Nigeria identified factors that determine the practices of EBF to include being unemployed, age of infants less than two months, full-time housewives, vaginal birth, delivery at a health facility, and mothers without breast complications [8, 13]. Although there are studies on breastfeeding practices in Nigeria, there is limited information on the determinants of EBF, which should form the focus of future interventions [9,13]. We, therefore, hypothesize that the prevalence of EBF practices and its determinants may differ from other studies. Hence, this study assessed the EBF practices and its determinants among mothers of infants less than six months attending an immunization clinic at a Teaching Hospital in Southwest Nigeria.

## Materials and methods

Study design and setting

This was a cross-sectional descriptive study carried out between 20th May 2020 and 25th June 2020 among mothers/caregivers of children aged less than six months that brought their children for immunization at the Bowen University Teaching Hospital (BUTH), Ogbomoso. The BUTH immunization clinic is held twice weekly and has an average attendance of 60-80 patients in a week. The immunization clinic also serves as the growth monitoring and the well-baby clinic where anthropometric measurements of the children are taken and plotted on the baby’s growth chart. When a child was noticed to be deviating from his growth pattern, he/she was referred to the paediatric clinic for further evaluation.

Study participants

The inclusion criteria were mothers/caregivers who consented to take part in the study, and whose children were between 0 and less than six months. We excluded mothers/caregivers of children older than 6 months, too sick to receive immunization and those whose children were referred to the paediatric clinic for further evaluation.

The recruitment of the eligible mothers was based on convenience sampling technique and involved consecutive consenting mothers till the sample size was attained.

Sample size determination

We estimated the minimum sample size required for the study using Cochran's formula. A standard normal deviate of 1.96 was used and the prevalence of children less than six months who received exclusive breastfeeding was assumed to be 19% based on results from a previous study from the Southwest region of the county [14], at a tolerable margin of error of 5%. A 10% non-response rate was anticipated and corrected for in estimating the minimum sample size.

Data collection method and instrument

A semi-structured, interviewer-administered questionnaire which was developed by reviewing extant literature was used to get relevant history and socio-demographic information from the participating mothers/caregivers. The instrument also collected data on nutritional history of each infant, including 24-hour dietary/breastfeeding recall and other breastfeeding practices since the child’s birth. It was interpreted into the Yoruba language for those who prefer to respond in their local language. A back translation into English language was done to preserve the original meanings of the questions asked. The meaning of exclusive breastfeeding was also explained to them in plain language.

Four nurses working in the Paediatric Department of BUTH assisted in data collection after they were trained by the principal investigator on questionnaire administration to mothers. The training was conducted within two days and involved practical simulations.

Pretesting of Questionnaire

The questionnaires were pretested among 30 mothers at the paediatric outpatient clinic of BUTH, Ogbomoso before the commencement of the study. This was a different department from the site used for the main study. The exercise helped to assess consistency, adequacy, and appropriateness of the questions to strengthen internal validity of the instrument. Ambiguous or irrelevant questions were either modified or deleted based on the objectives of the study.

Definition of key variables

Exclusive Breast Feeding

We defined exclusive breastfeeding practice as a mother who fed only breast milk, and no other liquids or solids except for oral rehydration solution, supplements, or medicines to her child aged less than six months since birth [3].

Social Class

The social class of the family was determined using the Oyedeji [15] classification of social class, which is based on mother’s and father’s occupation and educational attainment. The first and second social classes were regrouped as the upper social class, the third-social class as the middle class, while the fourth and fifth social classes were regrouped as the lower social class.

Outcomes Measured

The primary outcome measured was the prevalence of exclusive breastfeeding practice among the mothers of infants less than six months. The secondary outcome of this study was the determinants of EBF practices among mothers.

Ethical consideration

Ethical approval (approval no: BUTH/REC-119) for the study was obtained from the Bowen University Teaching Hospital Health Research Ethics Committee (HREC). Consent was sought from the eligible mothers/caregivers after a clear explanation of the study was given to them by the researchers. Participation was entirely voluntary, and respondents were free to opt out at any stage of the interview if they so wished. Confidentiality of information received from respondent was assured as the questionnaire was made anonymous and data entered into a computer which was only accessible to the principal investigator. Children observed to have symptoms and signs of malnutrition were referred to the paediatric department of BUTH for further evaluation.

Statistical analysis

Filled questionnaires were edited daily for completeness before data entry was done. Data analysis was carried out using the IBM Statistical Package for Social Sciences (SPSS) version 23.0 (IBM Corp., Armonk, NY) for windows. Mean and standard deviation were used as summary statistics for continuous variables while categorical variables were summarized using percentages and presented in Tables and Charts. The infants' age was not normally distributed and was summarized as median with interquartile range (IQR). Chi square test was used at the bivariate level to compare categorical variables. A Stepwise Binary Logistic regression was built at the multivariate level. Variables imputed into the model were selected based on whether they were statistically significant at bivariate level or whether they had been reported in previous studies as significant predictors good EBF practices. Adjusted Odds Ratio and Confidence Intervals were computed to evaluate factors which were significant determinants of good EBF practice among study participants. The level of statistical significance was set at p < 0.05.

## Results

A total of 300 questionnaires were administered but 271 of them were returned satisfactorily completed (response rate of 90.3%). As shown in Table 1, the mean age ±SD of the mothers was 30.4 ± 5.0 years. Most (49.1%) of the mothers belonged to the age group 30-39 years. Most of the mothers had a post-secondary level of education (177; 65.3%). Also, most of the children were from a monogamous family system (262, 96.7%). Table 1 shows other details of maternal characteristics.

**Table 1:** General characteristics of the mothers BUTH: Bowen University Teaching Hospital; ANC: Antenatal clinic

Variables	Frequency	Percent
Age of the mother (years)		
<20	1	0.4
20-29	126	46.5
30-39	133	49.1
40-49	10	3.7
50-59	1	0.4
Mean age: 30.4 ± 5.0 years		
Educational status of the mother		
Primary	5	1.9
Secondary	89	32.8
Post-secondary	177	65.3
Social class		
Lower	8	3.0
Middle	214	79.0
Upper	49	18.0
Religion of the mothers		
Christianity	227	83.8
Islam	44	16.2
Type of family		
Monogamous	262	96.7
Polygamous	9	3.3
Family size		
4 and below	184	67.9
More than 4	87	32.1
ANC attendance		
Yes	237	87.5
No	34	12.5
Parity		
1	119	43.9
2-4	150	55.4
> 4	2	0.7
Mode of delivery		
Vaginal delivery	183	67.5
Caesarean section	88	32.5
Place of delivery		
Government hospital	93	34.3
Mission Hospital (BUTH)	93	34.3
Private hospital	74	27.3
Home delivery	11	4.1

The median (interquartile range) age of the infants was 1.5 (0.5-3.0) months. Amongst the infants, males were 137 (50.6%) as shown in Table 2.

**Table 2:** General characteristics of the infants

Variables	Frequency	Percent
Age (month)		
0.0-1.0	104	38.4
1.1-2.0	77	28.4
2.1-3.0	49	18.1
3.1-4.0	27	10.0
4.1-5.0	9	3.3
5.1-6.0	5	1.8
Gender of child		
Male	137	50.6
Female	134	49.4
Birth order		
1^st^	99	36.6
2^nd^-4^th^	167	61.6
More than 4^th^	5	1.8

**Figure 1:**
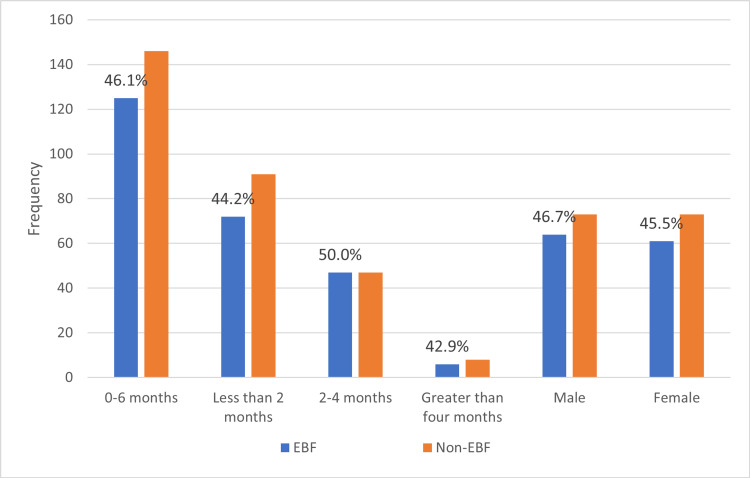
Prevalence of exclusive breastfeeding (EBF) and non-EBF.

The overall prevalence of exclusive breastfeeding (EBF) from infants (0-6 months) was 46.1% (125/271). The highest prevalence was in children aged two to four months (47/94; 50.0%.) as shown in Figure 1.

**Figure 2:**
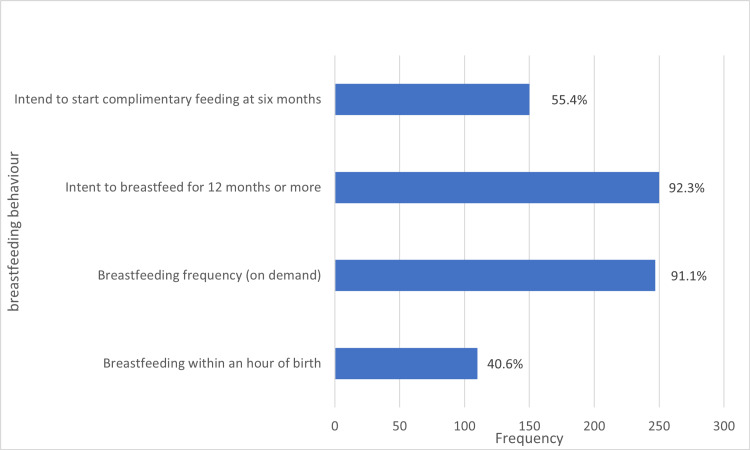
Breastfeeding behaviour and practice among the respondents.

Based on the good breastfeeding practices, 40.6% of mothers breastfed their infants within an hour of birth, with most breastfeeding on demand (91.1%) as shown in Figure 2.

Among the mothers who did not practice EBF to their infants, the most common reason given was maternal illness (25/146, 17.0%) while only a mother mentioned fear of her child becoming addicted to breastfeeding (1/146, 0.7%) as the main reason for not practicing EBF. This is shown in Table 3.

**Table 3:** Reasons for non-exclusive breastfeeding (n = 146)

Variables	Frequency	Percent
The baby continued to be hungry after breastfeeding	7	4.8
Maternal illness	25	17.1
Fear of baby becoming addicted to breastfeeding	1	0.7
Pressure from relative to give non-breast milk feeds	14	9.6
Need to resume for work	10	6.8
Pain in the breast	4	2.7

Among the maternal and infant variables, factors associated with EBF practices included mothers’ age > 30 years (OR 2.080, 95% CI 1.274, 3.395), family size greater than four (OR 2.425, 95% CI 1.438, 4.089) and having vaginal delivery (OR 2.810, 95% CI 1.634, 4.835). After controlling for confounders, only family size and having vaginal delivery were determinants of EBF with adjusted OR (95% CI) of 2.053 (1.120, 3.762), and 2.767 (1.585, 4.829), respectively (Table 4).

**Table 4:** Determinants of EBF practice among mothers with infants less than six months (n = 271) EBF: Exclusive breastfeeding; Mothers’ Educ: mothers’ educational level; grp: group

Variables	Categories	EBF (%)	Non-EBF (%)	Unadjusted		Adjusted	
				Odds ratio	95% CI	Odds ratio	95% CI
Mothers’ age grp	≤ 30 years	60 (38.5)	96 (61.5)	1			
	> 30 years	65 (56.5)	50 (43.5)	2.080	1.274, 3.395	1.431	0.809, 2.530
Mothers' Educ.	Primary	2 (40.0)	3 (60.0)	1			
	Secondary	44 (49.4)	45 (50.6)	1.209	0.197, 7.415		
	Post-secondary	79 (44.6)	98 (55.4)	0.824	0.495, 1.373		
Socio-economic class	Upper	29 (59.2)	20 (40.8)	1			
	Middle	94 (43.9)	120 (56.1)	0.230	0.042, 1.257		
	Lower	2 (25.0)	6 (75.0)	0.426	0.084, 2.157		
Mothers’ religion	Christianity	104 (45.8)	123 (54.2)	1			
	Islam	21 (47.7)	23 (52.7)	1.080	0.566, 2.062		
Family type	Monogamous	120 (45.8)	142 (54.2)	1			
	Polygamous	5 (55.6)	4 (44.4)	1.479	0.388, 5.632		
Family size	≤ 4	72 (39.1)	112 (60.9)	1			
	> 4	53 (60.9)	34 (39.1)	2.425	1.438, 4.089	2.053	1.120, 3.762
ANC attendance	Yes	108 (45.6)	129 (54.4)	1			
	No	17 (50.0)	17 (50.0)	1.194	0.582, 2.452		
Parity	1	34 (28.6)	85 (71.4)	1			
	2-4	90 (60.0)	60 (40.0)	2.5	0.152, 41.120		
	> 4	1 (50.0)	1 (50.0)	0.667	0.041, 10.865		
Mode of delivery	Cesarean section	26 (29.5)	62 (70.5)	1			
	Vaginal delivery	99 (54.1)	84 (45.9)	2.810	1.634, 4.835	2.767	1.585, 4.829
Place of delivery	Govt. hospital	40 (43.0)	53 (57.0)	1			
	BUTH	42 (45.2)	51 (54.8)	2.319	0.635, 8.468		
	Private hospital	36 (48.7)	38 (51.3)	2.125	0.582, 7.755		
	Home delivery	7 (63.6)	4 (36.4)	1.847	0.498, 6.848		
Infants’ gender	Male	64 (46.7)	73 (53.3)	1			
	Female	61 (45.5)	73 (54.5)	0.953	0.591, 1.537		
Birth order	1^st^	22 (22.2)	77 (77.8)	1			
	2^nd^-4^th^	101 (60.5)	66 (39.5)	2.333	0.367, 14.852		
	> 4^th^	2 (40.0)	3 (60.0)	0.436	0.071, 2.678		

## Discussion

Exclusive breastfeeding remains a cost-effective means of improving the childhood health indices, especially in the LMICs, where there is limited access to health care. This study assessed the prevalence of EBF practice and its determinants among mother-infant pairs at BUTH, Ogbomoso, Southwestern Nigeria. Our study showed a prevalence of EBF of 46.1%. The prevalence of EBF is higher than the national average reported in the 2018 NDHS [12]. This may be because the current study was hospital-based, as opposed to a community survey in the NDHS. Besides, about two-thirds of the mothers recruited in this study have at least a post-secondary education, which might have correlated positively to a better understanding of the widely publicised infant and young child feeding practices. The association between exclusive breastfeeding and maternal education has been previously reported by Adewuyi and Adefemi [16]. The highest rate of EBF (50.0%) in this study was in the 2-4 months age-group. This contradicts earlier reports that the rate of EBF decreases as the infant's age increases in Nigeria [16]. The explanation for the observed low EBF practice within the first month of life could be due to the erroneous traditional belief by many Nigerian mothers that initial breast milk (particularly colostrum) is unhealthy for the baby [9, 16]. Similarly, lower EBF practices after the fourth month of life could be due to the widespread myth that breast milk only is no longer sufficient to meet the nutritional demands of babies beyond the first three months of life [14].

Nine out of ten of the children were breastfed on demand; however, only 40% of the infants were put to the breast within an hour of birth, which is consistent with other studies [9, 17, 18]. In contrast, a much higher number of mothers (53%) breastfed their children within an hour of birth in Sokoto, North-west Nigeria [9]. The findings of a low level of early initiation of breastfeeding in this study underscores the need for training and retraining of health workers who are present in the delivery room for helping mothers to start breastfeeding immediately after birth. It also brings to the fore the need for more community awareness/health education on appropriate breastfeeding practices such as early initiation of breastfeeding. This will ensure that babies commence breastfeeding early in their life with the associated many benefits including the transfer of protective factors from mother to child through the colostrum, skin-to-skin contact between mother and child, which helps regulate new-borns’ body temperature and exposes them to beneficial bacteria from their mother’s skin [4, 7].

Mothers’ age greater than 30 years, vaginal delivery, and family size greater than four were factors associated with EBF. However, only vaginal delivery and family size greater than four were the determinants of EBF. In contrast, a study in Nigeria by Ihudiebube-Splendor et al., identified the absence of breastfeeding problems and mass media as a source of information (on breastfeeding) as the determinants of EBF [19]. In Ethiopia, the determinants of EBF were mothers of infants less than four months, vaginal births, married mothers, delivery at the hospital, and absence of breast complications [13]. The findings of vaginal delivery as a determinant of EBF may be related to the absence of post-operative pains and recovery from anesthesia seen in mothers who had Caesarean delivery. Post-operative pains are a recognized disruptor of lactogenesis which may affect EBF. Besides, mothers who have vaginal deliveries have early hospital discharges and bonding, which will encourage them to continue breastfeeding. The family size greater than four as a determinant of EBF may be because of better knowledge on importance of breastfeeding, mothering experience, and possibly earlier post-partum recovery. In Enugu, Southeast Nigeria, Ihudiebube-Splendor et al. [19] observed inadequate breastfeeding knowledge among primiparous women.

The commonest reason for non-EBF identified in this study was maternal illness. Of note is the proportion of women that could not practice EBF because they had to resume work. The fact that employed women on maternity leave must resume work a few months (often three months) post-partum poses a significant threat to the continuation of EBF after three months of the baby's life [10, 14, 20]. Despite their profound knowledge on the benefits of good EBF practices, Sadoh et al. [21] reported that only 11.1% of the surveyed female medical doctors in Edo State of Nigeria practiced EBF for up to six months because they had to resume work. Furthermore, most organizations in Nigeria either have no breastfeeding supporting policies or designated facilities for nursing mothers to breastfeed their babies while at work. Osibogun et al. [10], in a study of female bankers in Lagos, Nigeria reported that less than 10% of the respondents had workplace support for breastfeeding. Similarly, Soomro et al. [22] reported that only 15% of the workplaces surveyed allowed breastfeeding breaks to working mothers. This is contradicting to the WHO recommendation and National Policy on infants and young child feeding in Nigeria. Thus, there is a need for improved work-based breastfeeding support for nursing mothers to enable them to practice EBF.

Some indicators of infant and young child feeding practices (IYCF) used in this study relied on historical recall and so may be affected by recall bias. Furthermore, the participants were drawn from the attendees at the immunization clinic and may not be an accurate reflection of the actual practices of breastfeeding in the community.

## Conclusions

This study showed an average level practice of EBF, and one in two mothers practices EBF to their infants in the first six months of life. In addition, only two out of five babies commenced breastfeeding within an hour of birth. Factors associated with EBF include mothers' age greater than 30 years, vaginal delivery, and family size of more than four. Only vaginal delivery and family size greater than four were the determinants of EBF. The most common reason for non-EBF was maternal illness and the need to resume work. Authors, therefore, recommend that the practice of EBF needs to be protected, promoted, and supported for optimal growth and development of Nigerian infants. In line with the Baby-Friendly Hospital Initiative, all healthcare facilities where deliveries occur should be assisted to implement the ‘Ten Steps to Successful Breast Feeding’.

## Appendices

STUDY PROFORMA

INFANT AND YOUNG CHILD FEEDING PRACTICES AMONG MOTHERS OF CHILDREN 0-6 MONTHS OLD ATTENDING THE IMMUNISATION CLINIC OF THE BOWEN UNIVERSITY TEACHING HOSPITAL, OGBOMOSO

Serial no .: ____________________

A. Biodata

Age in months:________________ Date of birth_______________ Gender ___________

Age of respondent__________ Relationship to child________________________

1.      Educational status of father i. primary ii. Secondary iii. Post-secondary iv. None v. others, pls state___________________

2.      Occupation of father ___________________

3.      Educational status of mother i. primary ii. Secondary iii. Post-secondary iv. None v. others, pls state___________________

4.      Occupation of mother ___________________

5.      Tribe of father __________________________

6.      Tribe of mother _________________________

7.      Mother’s age ___________________________

8.      Religion_______________________________

9.      Family size (total of mother, father, number of children) ________________

10.  Family type a. Monogamous b. polygamous c. others, pls specify _______________

11.  Position of child in the family ____________________

12.  Income of family/month____________________

a. N1000-N10,000

b. N10,000-N50,000

c. N50,000-N100,000

d. N>100,000

13.  Where do you live? (in/around Ogbomoso) __________________________

B. Mother’s history

1. Parity of mother _______

2. Antenatal clinic attended during this child’s pregnancy. Yes_______ No____

3. If yes to question 2 above, where?______________________

4. How many ANC visits did you have during the pregnancy? a. 1-3 visits b. >/3 visits

5. Child delivered where? (Govt hospital/private hospital/Home/mission house/TBA)    ________________________

If at home, supervised by who? __________________

6. Mode of delivery (Caesarean section, vaginal delivery)_____________________________

7. Did you receive any counselling on infant and young child feeding during ANC visits? Yes_______ No____

8. Did you receive any counselling on infant and young child feeding during postnatal or immunisation visits? Yes_______ No____

9. Availability of home garden Yes_______ No____

C. Feeding practices

1)      Was this child ever breastfed? Yes______ No______

2)      When was the child first put to breast, either mother was lactating or not? A. Within the first hour of life b. after the first hour of life but within 24 hours of life c. after 24 hours of life

3)      What were the things put in his mouth first 3 days of life? (water, herbal concoction, infant formula, honey etc) ____________________________

4)      Was the child exclusively breastfed in the first 6 months of life? Yes______ No______

5)      If no to question 3 above, at what age was he commenced on

i. other feeds?_________

ii. water? _________

5. Mode of feeding in the first 6 months of life

a. breast milk only

b. breast milk and water

c breast milk in addition to infant formula

d. breast milk in addition to pap

e. breast milk in addition to other foods

6. Is the child still breastfeeding? Yes _____ No ___

7. If no to question 6 above, at what age was child completely weaned off breast milk?

8. Is the child on bottle feeding (feeder)? Yes _____ No ___

9. How many times was the child breastfed in the last 24 hours?

a. < 6 to 8 times

b. 6 to 8 times

c. > 8 times

d. As often as the baby wants

10. How long does the child breastfeed for?

a. Less than half an hour

b. Half an hour

c. More than half an hour

d. I don't count

e. Breastfeeding till when asleep

11. When do you intend to start other foods for the child? (at what age)_______________

12. When do you intend to stop breastfeeding totally (age of child in months)

 a. ≥ 6

   b. 7 to 12

   c. 13 to 18

 d. 19 to 24

13.  What other meal (food) did the child eat in the last 24 hours? ____________

14. If child not only on breast milk, what is the reason?  

a. the perception that babies continued to be hungry after breastfeeding

b. maternal health problems. if mother was sick, what illness________

c. fear of babies becoming addicted to breast milk 

d. pressure from mother-in-law /other relatives

e. pains in the breast /nipples

f. the need to return to work

g. other reasons, pls state _________________________________

D. Anthropometry

Weight /kg ____________     Length/cm ____________

MUAC /cm ___________     Head circumference________________cm
